# Molecular correlates and therapeutic targets in T cell-inflamed versus non-T cell-inflamed tumors across cancer types

**DOI:** 10.1186/s13073-020-00787-6

**Published:** 2020-10-27

**Authors:** Riyue Bao, Daniel Stapor, Jason J. Luke

**Affiliations:** 1 Hillman Cancer Center, UPMC, Pittsburgh, PA 15232 USA; 2 Department of Medicine, University of Pittsburgh, Pittsburgh, PA 15232 USA

**Keywords:** T cell-inflamed, Immune evasion, Genomics, Transcriptomics, TCGA

## Abstract

**Background:**

The T cell-inflamed tumor microenvironment, characterized by CD8 T cells and type I/II interferon transcripts, is an important cancer immunotherapy biomarker. Tumor mutational burden (TMB) may also dictate response, and some oncogenes (i.e., WNT/β-catenin) are known to mediate immunosuppression.

**Methods:**

We performed an integrated multi-omic analysis of human cancer including 11,607 tumors across multiple databases and patients treated with anti-PD1. After adjusting for TMB, we correlated the T cell-inflamed gene expression signature with somatic mutations, transcriptional programs, and relevant proteome for different immune phenotypes, by tumor type and across cancers.

**Results:**

Strong correlations were noted between mutations in oncogenes and tumor suppressor genes and non-T cell-inflamed tumors with examples including *IDH1* and *GNAQ* as well as less well-known genes including *KDM6A*, *CD11c*, and genes with unknown functions. Conversely, we observe genes associating with the T cell-inflamed phenotype including *VHL* and *PBRM1*. Analyzing gene expression patterns, we identify oncogenic mediators of immune exclusion across cancer types (HIF1A and MYC) as well as novel examples in specific tumors such as sonic hedgehog signaling, hormone signaling and transcription factors. Using network analysis, somatic and transcriptomic events were integrated. In contrast to previous reports of individual tumor types such as melanoma, integrative pan-cancer analysis demonstrates that most non-T cell-inflamed tumors are influenced by multiple signaling pathways and that increasing numbers of co-activated pathways leads to more highly non-T cell-inflamed tumors. Validating these analyses, we observe highly consistent inverse relationships between pathway protein levels and the T cell-inflamed gene expression across cancers. Finally, we integrate available databases for drugs that might overcome or augment the identified mechanisms.

**Conclusions:**

These results nominate molecular targets and drugs potentially available for further study and potential immediate translation into clinical trials for patients with cancer.

## Background

Based on the hypothesis of immuno-editing, a small number of early tumors persist and eventually progress to metastatic disease [1]. During the time from tumor formation to systemic spread, genomic and antigenic editing defines a tumor-immune microenvironment that facilitates the metastatic process [1]. Immune profiling of tumor lysates from patients receiving cancer vaccines suggested a paradigm of two broad phenotypes characterized by the presence or absence of CD8+ effector tumor-infiltrating lymphocytes (TIL) and other mediators of an adaptive immune response [2]. These tumors have been described as T cell-inflamed and demonstrate a transcriptional profile driven by type I interferons (IFNs) as well as expression of many IFN-γ linked immunosuppressive mechanisms [3]. This is in contrast with non-T cell-inflamed tumors, which have low TIL count and a minimal inflammatory signature. The interaction of the host immunity with tumor cells has been identified as a hallmark of cancer based on the rapid development of immune-checkpoint blocking immunotherapy [4], predominately centered on programmed cell death protein 1 (PD1) [5]. While multiple biomarkers have been advanced to better describe the activity of checkpoint immunotherapy, notably tumor mutational burden (TMB), a biological predictor to an anti-cancer immune response is the elaboration of IFN-γ and the development of T cell-inflamed tumor microenvironment. Given this, the T cell-inflamed phenotype may be leveraged to study factors promoting or limiting a productive anti-tumor immune response.

An aspect impacting the efficacy of cancer immunotherapy may be tumor- or tumor microenvironment-intrinsic molecular factors. While immuno-editing may shape the overall antigenic landscape of a tumor, pressure from the immune response will additionally select for tumor clones with specific mutations and/or dysregulation of gene expression pathways that mediate immune evasion. The first tumor-intrinsic oncogene linked to immune-exclusion was β-catenin in the context of metastatic melanoma [6]. Activation of the WNT/β-catenin pathway, by multiple mechanisms, eliminated required chemokine gradients for the recruitment of BATF3 lineage dendritic cells, with a functional impact of resistance to both checkpoint blocking and adoptive cellular immunotherapy [6, 7]. Subsequently, this impact of β-catenin has been observed as a mechanism of immuno-suppression across many cancer types [8]. A growing list of molecular alterations beyond β-catenin is also being recognized to facilitate immune evasion including PTEN loss or MYC pathway activation [9] with suggestions from prior reports that these pathways drive non-T cell-inflamed tumors independently [10]. Previous studies have expanded the number of somatic events associated with immune exclusion via computational approaches [11, 12] and identified transcriptional signatures that nominate regulators of checkpoint blockade response in patients [13], however, have yet to integrate somatic mutations and tumor oncogenic transcriptional pathway activation into the overall model.

Using a model wherein the T cell-inflamed tumor microenvironment is considered as a biological approximation of immunotherapy treatment response, we investigated molecular patterns that associate with the presence or absence of this phenotype with the idea that they may lead to therapeutic opportunities. We present a comprehensive genomic atlas of mutations, gene expression patterns, and network analyses to nominate molecular aberrations associated with the presence or absence of the T cell-inflamed tumor microenvironment in tumor-type specific and pan-cancer fashion. Integration of pathway modeling and network analysis across cancer types challenges previous models of tumor-intrinsic signaling on immuno-suppression. Rather than individual pathways being dominant, a more complex model in which signaling pathways interact with each other and combine to drive progressively more non-T cell-inflamed tumors appears likely. Finally, we detail available therapeutics that could be explored to reverse or augment immune phenotypes. These data suggest next steps in translational and clinical research and provide a more nuanced model of tumor-intrinsic signaling for future investigation.

## Methods

### Datasets in this study

Datasets used in this study include (Additional file 1: Table S1): (1) The Cancer Genome Atlas [14] (TCGA) (*n* = 9244) (somatic mutations, RNAseq gene expression, reverse phase protein array (RPPA) protein abundance, and clinical data), (2) International Cancer Genome Consortium [15] (ICGC) (*n* = 1161) (RNAseq gene expression), (3) Clinical Proteomic Tumor Analysis Consortium [16, 17] (CPTAC) (*n* = 870) (RNAseq gene expression), (4) 500 cancer patients diagnosed with metastatic cancers (MET500) [18, 19] (*n* = 259) (RNAseq gene expression), and (5) metastatic melanoma patient cohort treated with anti-PD1 [20, 21] (*n* = 73) (RNAseq gene expression and clinical data). Further details about each specific dataset are described below in each relevant analysis section.

### TCGA cancer datasets

Level 3 RNA-seq gene expression (release February 4, 2015) and level 4 somatic mutations (release January 28, 2016) were downloaded for 31 solid tumor types from TCGA preprocessed by Broad Institute’s team [14]. Clinical data were downloaded from standardized clinicopathologic annotation data tables in TCGA Pan-Cancer Clinical Data Resource (TCGA-CDR) [22]. Gene expression was quantified by RNA-Seq by Expectation Maximization (RSEM) algorithm [23], with raw read counts mapped to gene features. The count-based gene expression was normalized across all samples using the upper quartile method, followed by log_2_ transformation. A total of 9555 tumor samples were initially downloaded. Lymphoid neoplasm diffuse large B-cell lymphoma (DLBC), acute myeloid leukemia (LAML), and thymoma (THYM) were excluded from analysis because only 0 to 2 tumors were classified as the non-T cell-inflamed group in those tumor types (Additional file 1: Table S2) and therefore do not provide adequate sample size for the group-wise comparison contrasting T cell-inflamed versus non-T cell-inflamed groups. A total of 9244 tumors were included in the analysis, consisting of 8890 primary tumors from 30 tumor types and 354 metastatic samples from one tumor type (skin cutaneous melanoma (SKCM)).

### Identification of non-T cell-inflamed and T cell-inflamed tumor groups

The tumor samples were categorized into non-T cell-inflamed, T cell-inflamed, and intermediate groups using a defined T cell-inflamed gene expression signature consisting of 160 genes, the same as previously described [24]. In brief, the normalized gene expression was transformed into a scoring system where each gene is defined as upregulated (+ 1), downregulated (− 1), or unchanged (0) relative to the mean of its expression across all samples. By adding scores of all 160 genes from the signature together, we obtained a sample-wise gene score ranging from − 160 (if all genes are downregulated) to + 160 (if all genes are upregulated). Tumors of score < − 80 were categorized as non-T cell-inflamed, those of score > + 80 as T cell inflamed, and the rest as intermediate. The list of 160 genes is provided in Additional file 1: Table S3.

### Differential gene expression detection and pathway activation prediction from RNAseq data

Within individual tumor type, the non-T cell-inflamed group was contrasted against the T cell-inflamed group and differentially expressed genes (DEGs) were identified by Linear Models for Microarray and RNA-Seq Data (limma) voom algorithm with precision weights (v3.38.3) [25]. The empirical quality weights were estimated to account for variation in precision among samples and were shown to increase the performance of DEG detection, especially for highly heterogeneous human tumor samples. Significant DEGs were filtered by FDR-adjusted *P* < 0.05 and fold change ≥ 1.5 or ≤ − 1.5. Pathways significantly affected by the DEGs were detected by IPA^®^ (QIAGEN Inc., Germany) with the curated Ingenuity Knowledge Base (accessed November 2015). Upstream transcriptional regulators and their change of direction were predicted based on the cumulative effect of downstream target molecules (encoded by DEGs) implemented in IPA^®^ causal network analysis [26]. Those of overlap *P* < 0.05 (measuring the enrichment of target molecules in the dataset) and activation *z*-score > 1.95 were selected for further analysis. The predicted upstream regulators were further annotated by Kyoto Encyclopedia of Genes and Genomes (KEGG) database and used to build a pathway-to-pathway network based on shared upstream regulators among annotated pathways in Cytoscape (v3.7.1) [27], with nodes denoted as individual KEGG signaling pathways, and edge size denoted as the number of shared upstream regulators between pathways.

### Differential somatic mutation enrichment detection from whole-exome sequencing data

For somatic mutations, those predicted to be altering protein sequences (referred to as, NSSMs, non-synonymous somatic mutations), including non-synonymous/stoploss/stopgain single nucleotide variants (SNVs), frameshift/non-frameshift small insertions and deletions (indels), and variants that affect the splicing site, were kept for analysis. TMB was defined as the total number of NSSMs per tumor. For each tumor sample, a binary label was assigned per gene to indicate if this tumor harbors NSSMs in this gene (Y, mutated) or not (N, non-mutated). The association between mutation status and tumor groups was tested using logistic regression models controlling for TMB. For each gene, a logistic regression model was fitted with tumor group (T cell-inflamed or non-T cell-inflamed) as the response variable and two explanatory variables (mutation status (Y or N, categorical variable), log_10_-transformed TMB (continuous variable)) using R function *glm* from R library stats (v3.6.1). *P* values of the “mutation status” term contrasting mutated versus non-mutated samples were extracted from the fitted models and subsequently corrected by Benjamini-Hochberg FDR (BH-FDR) method for multiple comparisons. For each test, genes mutated in < 5% of the samples from individual tumor type or < 0.5% across all samples were excluded from statistical testing in tumor type-specific analysis and pan-cancer analysis, respectively. To nominate a more comprehensive list of potentially relevant mutated genes associated with immune phenotypes, we used FDR cutoff of 0.10. In addition, we have calculated per-gene per-tumor type mutation frequency and made data files available at our GitHub repository [28].

### Prediction of mutation effect from non-synonymous somatic mutations

For variant functional prediction including loss of function (LOF) and gain of function (GOF), we annotated NSSMs using OncoKB [29] annotator (v2.2.0) (https://github.com/oncokb/oncokb-annotator) with two input files, a somatic mutation list and a clinical data table with manually inspected TCGA cancer ID to MSKCC-OncoTree cancer ID conversion (available at [30]), with program command as “MafAnnotator.py -i mutation_input -o annotation_output -c clinical_input -b oncokb_api_token -a.” In addition, we have annotated *TP53* mutations for all samples and made data files available at our GitHub repository [28].

### Assessment of tumor-specific pathway activation in external cancer genomic cohorts

The RNAseq gene expression data were downloaded from ICGC [15] (accessed February 11, 2020), CPTAC [16, 17] (accessed May 26, 2020), and MET500 [18, 19]. A total of 22 studies (2290 tumor samples total) from external cancer genomic cohorts were included in our analysis. After excluding tumor types of < 25 samples, the ICGC cohort used in this study is made up of 1161 samples from seven tumor types with one for breast (BRCA-KR), head and neck (ORCA-IN), kidney (RECA-EU), and ovarian (OV-AU) as well as two each for liver (LICA-FR and LIRI-JP), pancreatic (PACA-AU and PACA-CA) and prostate (PRAD-CA and PRAD-FR) cancers. Ewing sarcoma from ICGC (BOCA-FR) was excluded from our analysis, given the distinctly different clinical population relative to sarcoma from TCGA. Eight tumor types were included from CPTAC (BRCA, COAD, GBM, HNSC, KIRC, LUAD, OV, and UCEC), and four were included from MET500 (BRCA, CHOL, PRAD and SARC). For ICGC, preprocessed and normalized read count files were downloaded, and the values from the column “normalized_read_count” were used for analysis. For CPTAC, the harmonized read count files from Genomic Data Commons [31] (GDC) were downloaded and normalized across all samples using trimmed mean of *M* values (TMM) method and converted to counts per million (CPM). For MET500, raw RNAseq data in FastQ format were downloaded from dbGAP (accession number phs000673.v4.p1) and quantified for gene expression using Kallisto [32] (v0.44.0), summarized into gene level by tximport [33], followed by TMM normalization and CPM transformation. All expression values were log_2_-transformed before statistical analysis. For each pathway discovered to be activated in non-T cell-inflamed tumors in TCGA, the pathway gene expression was calculated by taking the median expression across all genes involved in the pathway. The pathway gene expression was correlated with T cell-inflamed gene expression using Pearson’s correlation. Similarly, Pearson’s correlation was calculated in TCGA samples for the same tumor-specific pathways in each tumor type. To assess the consistency of pathway gene expression and T cell-inflamed gene expression correlation between TCGA and external cancer genomic cohorts, for each tumor-specific pathway, we assigned a binary category as *negative* if it is inversely correlated with T cell-inflamed gene expression, and *positive* if it is positively correlated, and calculated Jaccard similarity coefficient using R function *jaccard* from R library jaccard (v0.1.0).

### Assessment of metastatic melanoma-specific pathways in a cohort of patients treated with anti-PD1

Raw RNAseq data in FastQ format were downloaded from a published metastatic melanoma cohort treated with anti-PD1 monotherapy or anti-PD1/anti-CTLA4 combination immunotherapy [20, 21] (SRA accession number PRJEB23709). Pre-treatment samples were included in our analysis (*n* = 73). Gene expression was quantified following the protocol that we previously published [34]. In brief, after quality assessment using FastQC [35] (v0.11.5), transcript abundance was quantified using Kallisto [32] (v0.43.1) with human reference transcriptome (GRCh38) and Gencode gene annotation (v28) and summarized into gene level by tximport [33] (v1.6.0) [33]. Lowly expressed genes were removed (defined as, CPM ≤ 3). Gene expression was normalized across all samples by TMM method, and log_2_ transformed. For each pathway discovered to be activated in non-T cell-inflamed tumors in metastatic melanoma from TCGA including CTNNB1 and KLF4, the pathway gene expression was calculated by taking the median expression across all genes involved in the pathway. The pathway gene expression was correlated with T cell-inflamed gene expression across all pre-treatment samples using Pearson’s correlation and compared between non-responders (NR, including progressive disease (PD) and stable disease (SD)) (*n* = 33) and responders (R, including partial responder (PR) and complete responder (CR)) (*n* = 40) using two-sided Welch Two Sample *t*-test.

### RPPA data analysis

Level 3 RPPA antibody-level protein abundance data (release January 28, 2016; patch July 14, 2016) produced by MD Anderson Cancer Center were downloaded from TCGA preprocessed by Broad Institute’s team [14] (accessed April 26, 2018). For each protein, its abundance was estimated using median-centered normalized values corresponding to the antibody. For CTNNB1, its protein level was matched corresponding to antibody “beta-Catenin” from the data files without gene annotation, and the gene-annotated RPPA data files were not used because of potential gene symbol mismatch for β-catenin. For all other molecules, the gene-annotated RPPA data files were used. Within each tumor type, a two-sided Pearson’s correlation test (R function *cor*.*test*) was performed between the abundance of a protein and T cell inflamed gene expression from normalized RNA-seq data, followed by BH-FDR correction for multiple testing.

### Assignment of NSSM score and pathway score to tumor samples

In the non-T cell-inflamed tumors, each sample was assigned an NSSM score representing the number of genes carrying NSSMs in this sample, and a pathway score representing the number of pathways activated in this sample. Then, the samples were grouped into 11 categories of NSSM scores and 11 categories of pathway scores each. NSSM score category 0 to 10 represents the samples harboring 0 to 10 co-mutated genes out of the 29 genes significantly enriched in non-T cell-inflamed tumors. For samples having more than 10 co-mutated genes, they were collapsed into the NSSM score category 10. Pathway score category 0 to 10 represents the samples with 0 to 10 co-activated pathways out of 266 pathways significantly activated in non-T cell-inflamed tumors. For samples having more than 10 co-activated pathways, they were collapsed into the pathway score category 10. The percentage of samples in each NSSM or pathway score category per tumor type was calculated.

### Heterogeneity in the distribution of mutations or pathway activation between tumor types

The method to assess the heterogeneity in the distribution of mutations or pathway activation between tumor types is illustrated as follows. The same analysis was performed for NSSM score categories and pathway score categories; therefore, we will take the NSSM score analysis as an example to describe the process. First, the percentage of samples in each NSSM score category in the non-T cell-inflamed tumors was calculated per tumor type, as described above (step 1). Then, the arithmetic mean percentage of samples in each NSSM score category was calculated across all tumor types, generating the mean distribution of NSSM score categories. For each tumor type, the Euclidean distance was computed between two vectors of numbers, the ones from the percentage of samples falling into each NSSM score category in this tumor type and the ones representing the mean distribution of NSSM score categories across all tumor types, hereby representing the deviation of NSSM score distribution of this tumor type from the mean distribution. This process was repeated for every tumor type, generating one Euclidean distance metrics per tumor type (hereafter referred to as, the NSSM score distances). Similarly, the same analysis was performed for pathway scores, generating the pathway score distances (step 2). Lastly, NSSM score distances and pathway score distances were compared to test if those are from the same distribution using two-sided two-sample Kolmogorov-Smirnov test (step 3). Significant differences indicated that the distribution of NSSM score distances was different from that of the pathway score distances, further suggesting different degrees of heterogeneity among tumor types between mutations and pathways.

### Modeling the relationship between T cell-inflamed gene expression and increasing number of co-mutated genes or co-activated pathways in tumor

The relationship between T cell-inflamed gene expression and NSSM or pathway score (described above) was modeled at per sample level using linear regression (R function *lm*). Score categories with ≥ 10 samples were included. NSSM score category 0 to 8 and pathway score category 0 to 33 were kept for further analysis. In this analysis, different from the analysis described above, samples with more than 10 co-activated pathways were kept as is and not collapsed into category 10. For each score category, the median of T cell-inflamed gene expression was calculated across samples in this category, and then fitted against the NSSM or pathway scores using formula *Expression* = *β*_0_ + *β*_1_ × *Score*, with *β*_0_ as the interception and *β*_1_ as the coefficient. Data were also fitted using polynomial regression models *Expression* = *β*_0_ + *β*_1_ × *Score* + *β*_2_ × *Score*^2^ and *Expression* = *β*_0_ + *β*_1_ × *Score* + *β*_2_ × *Score*^2^ + *β*_3_ × *Score*^3^, but those models were not significantly better than the linear regression model (*P* > 0.05, likelihood ratio test); hence, the first model was kept for final analysis.

### Drug-gene interaction and druggable gene category identification

Candidate genes identified by pathway activation or somatic mutation enrichment analysis were queried in The Drug Gene Interaction Database [36] (DGIdb, http://www.dgidb.org) (v3.0, accessed June 20, 2020) for gene-drug interactions (inhibitor, activator, etc.) and druggable gene categories (clinically actionable, drug resistance, etc.) with default settings. The DGIdb integrates resources from 20 databases and consists of existing drugs including those that are FDA-approved, antineoplastic, and/or immunotherapies.

### Statistical analysis

For analysis of the association between mutated genes and tumor groups, logistic regression was used with TMB as a covariate. For analysis of contingency tables including comparison of sample frequency between groups activated by pathways, two-sided Fisher’s exact test was used. Two-sided Welch Two Sample *t*-test was used to compare individual pathway gene expression between groups. Gene expression comparison between tumor groups was performed using linear regression models implemented in limma voom with precision weights. For multiple comparisons, *P* value was adjusted using BH-FDR correction for multiple testing [37]. Pearson’s correlation *r* was used for measuring statistical dependence between normalized and log_2_-transformed expression level of different genes. Linear regression was used to model the relationship between increasing number of co-mutated genes or co-activated pathways and T cell-inflamed gene expression in tumor. FDR-adjusted *P* < 0.10 was used for somatic mutation enrichment analyses; FDR-adjusted *P* < 0.05 was used for the gene expression and pathway analyses. Statistical analysis was performed using R (v3.5.1) and Bioconductor (v3.8).

## Results

### Candidate cancer oncogenes or tumor suppressor genes are rarely enriched in most cancer types between T cell-inflamed and non-T cell-inflamed tumors

Using a T cell-inflamed gene expression signature we previously described [24], we categorized tumor samples into non-T cell-inflamed, T cell-inflamed, and intermediate groups across 31 solid tumors from TCGA. To place our approach into clinical context, we correlated our T cell-inflamed gene expression signature with previously validated interferon-γ associated signatures including the immune cytolytic activity (CYT) [38] and the interferon gene expression profile (GEP) [39], observing Pearson’s correlation coefficients of 0.82 and 0.90 (*P* < 0.0001), respectively. To investigate the correlation between mutated genes and T cell-inflammation, we investigated 57 candidate cancer oncogenes or tumor suppressors documented in the mutational cancer drivers database IntOGen (05/2016 version) [40].

**Fig. 1:**
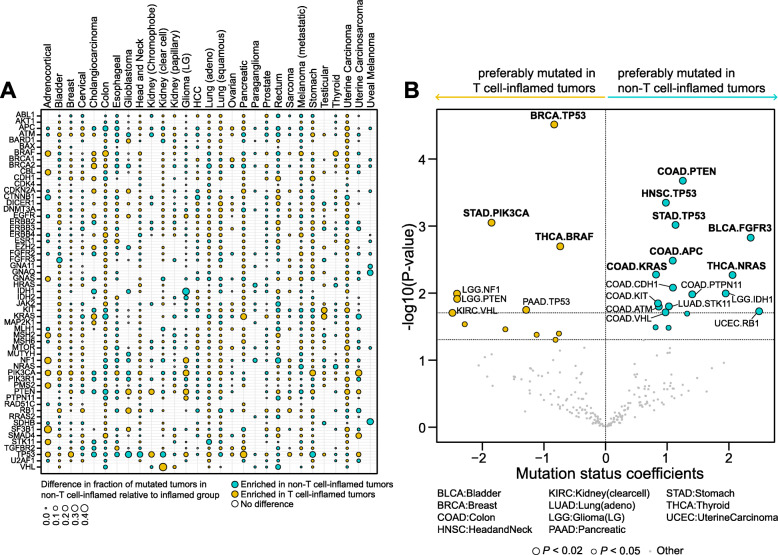
Cancer-specific relative enrichment of mutations in T cell-inflamed and non-T cell-inflamed tumors from known cancer driver genes. **a** Fifty-seven genes with mutation enrichment in one or more tumor types. Yellow and blue each represents genes preferably mutated in T cell-inflamed and non-T cell-inflamed tumors, respectively. Empty slots indicate a gene is not mutated in the tumor type. **b** Cancer-specific mutated genes that are preferably mutated in T cell-inflamed tumors (yellow) or non-T cell-inflamed tumors (blue). Coefficients of “mutation status” term from logistic regression models are shown on the *x*-axis, which describe the association between mutation status and tumor groups in the unit of logits. *P* values are shown on the *y*-axis. Each circle represents one gene in each tumor type. Out of 57 genes included in the analysis, those at FDR-adjusted *P* < 0.10 are shown in bold. For **b**, genes mutated in at least 5% of the samples in individual tumor types were included in statistical testing, hence shown on the volcano plots. Logistic regression with TMB as a covariate was used in **b**

For each gene, we compared the frequency of samples harboring NSSMs between non-T cell-inflamed and T cell-inflamed groups with relative enrichment of mutated samples shown in Fig. 1a. Given the growing clinical role of TMB in immunotherapy treatment, we incorporated this as a covariate in the logistic regression analysis, and in order to nominate a robust list of potentially relevant genes, we applied FDR-adjusted *P* < 0.10 for the somatic mutation enrichment analyses (see the “Methods” section). Twenty-nine tumor types with somatic mutation data available were included in the analysis (primary melanoma and mesothelioma did not have data available at the time of the study). Mutations preferentially found in T cell-inflamed tumors included *TP53* (breast cancer), *PIK3CA* (stomach), *BRAF* (thyroid), and *VHL* (kidney renal clear cell carcinoma) (Fig. 1b, left panel). In contrast, *FGFR3* (bladder), *TP53* (head and neck, stomach), *NRAS* (thyroid), and APC (colon) are among the genes more frequently mutated in non-T cell-inflamed tumors (Fig. 1b, right panel). After correction for multiple comparisons within the 57 genes and 29 tumor types investigated, we identified 10 genes with significant relative enrichment of mutated samples in T cell-inflamed or non-T cell-inflamed tumors from specific tumor types (Fig. 1b, bolded).

### Somatic mutations in oncogenic signaling pathways are enriched in non-T cell-inflamed tumors of individual cancer types

**Fig. 2:**
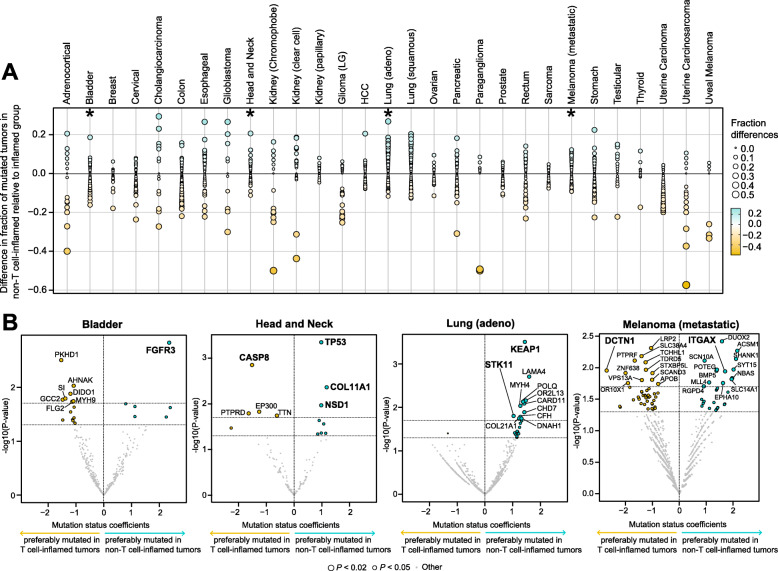
Cancer-specific relative enrichment of mutations in T cell-inflamed and non-T cell-inflamed tumors from all protein-coding genes on the genome. **a** Each tumor type shows a set of gene mutations enriched in T cell-inflamed or non-T cell-inflamed tumor group. Stars label the four representative tumor types in **b**. **b** Cancer-specific mutated genes that are preferably mutated in T cell-inflamed tumors (yellow) or non-T cell-inflamed tumors (blue) in four select tumor types. Coefficients of “mutation status” term from logistic regression models are shown on the *x*-axis, which describe the association between mutation status and tumor groups in the unit of logits. *P* values are shown on the *y*-axis. Individual genes of interest discussed in the main text were labeled in bold. The full list of genes with statistical metrics in the 29 tumor types is provided in Additional file 1: Table S4 and Additional file 1: Table S5. Each circle represents one gene in each tumor type. For **b**, genes mutated in at least 5% of the samples in individual tumor types were included in statistical testing, hence shown on the volcano plots. Logistic regression with TMB as a covariate was used in **b**

To probe molecular mediators of immune exclusion, we performed an unbiased genome-wide discovery of somatic mutations associated with T cell-inflamed or non-T cell-inflamed tumors within individual cancer types, with TMB as a covariate. We collapsed the NSSMs into gene level and counted the number of samples mutated in each of the 18,008 protein-coding genes (see the “Methods” section). The association between each mutated gene and tumor groups within individual cancer type was tested using logistic regression controlling for TMB. Each tumor type demonstrated a distinct mutation enrichment signature associated with T cell-inflamed or non-T cell-inflamed tumor microenvironment (Fig. 2a).

Four representative tumor types (urothelial, head and neck squamous, lung adenocarcinoma, and metastatic melanoma) selected based on the relevance of clinical immunotherapy are shown in Fig. 2b. In urothelial cancer, the gene differentially mutated most frequently in non-T cell-inflamed tumors is *FGFR3*, which was reported in our previous work [41]. In head and neck squamous carcinoma, *TP53* mutations are disproportionally mutated between the two groups, with a higher proportion of samples mutated in non-T cell-inflamed compared to T cell-inflamed tumors (82.6% versus 62.0%). In lung adenocarcinoma, *KEAP1* and *STK11* mutations are associated with the non-T cell-inflamed phenotype. Both genes are associated with resistance to PD1 blockade in lung cancer [42, 43]. In metastatic melanoma, *DCTN1* was found to be associated with the presence of T cell-inflammation while *ITGAX* (alias *CD11c*) is more frequently mutated in non-T cell-inflamed tumors. After FDR correction for multiple comparisons on all genes included in the analysis, among the mutations described above, *TP53* in head and neck squamous carcinoma remained significant (FDR-adjusted *P* = 0.035), others did not possibly due to sample size and an increased number of tests from the unbiased genome-wide approach than select cancer oncogenes or tumor suppressors genes. The full list of genes associated with the non-T cell-inflamed or T cell-inflamed phenotype in individual cancer types is provided in Additional file 1: Table S4 and Additional file 1: Table S5, respectively.

### Somatic mutations in oncogenic signaling pathways are shared in non-T cell-inflamed tumors across cancer types

**Fig. 3:**
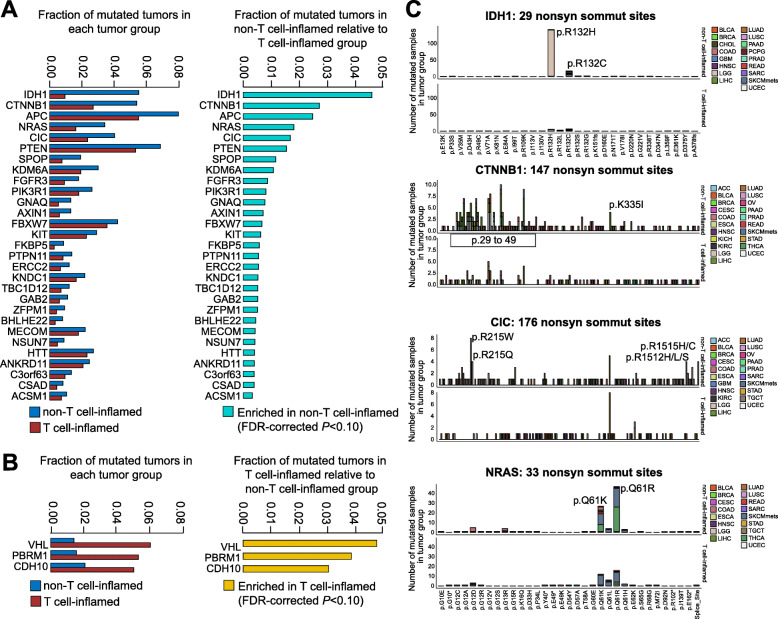
Pan-cancer relative enrichment of mutations in T cell-inflamed and non-inflamed tumors from all protein-coding genes on the genome. **a** Twenty-nine genes carrying NSSMs more frequently in non-T cell-inflamed relative to T cell-inflamed tumors at FDR-adjusted *P* < 0.10. **b** Three genes carrying NSSMs more frequent in T cell-inflamed relative to non-T cell-inflamed tumors at FDR-adjusted *P* < 0.10. The full list of genes with statistical metrics across all tumors is provided in Additional file 1: Table S6 and Additional file 1: Table S7. **c** Representative examples showing the protein-domain-specific enrichment of non-T cell-inflamed mutations in *IDH1*, *CTNNB1*, *CIC*, and *NRAS* pan-cancer wide. Individual amino acid changes are shown on the *x*-axis when space permits. The predicted mutation effects (loss of function (LOF), gain of function (GOF), etc.) of the 29 genes shown in **a** and three genes shown in **b** are provided in Additional file 1: Table S8 with summary statistics in Additional file 1: Table S9. For **a** and **b**, genes mutated in at least 0.5% of the samples pooled from all tumor types were included in statistical testing. Logistic regression with TMB as a covariate was used in **a** and **b**, with BH-FDR correction for multiple comparisons

To investigate shared mutations associated with immuno-suppression across multiple tumor types, we performed a pooled analysis of the 29 solid tumors with somatic mutation data available and identified NSSMs significantly enriched in T cell-inflamed or non-T cell-inflamed tumors. A total of 29 mutated genes were found to be significantly enriched in non-T cell-inflamed tumors at FDR-adjusted *P* < 0.10 in logistic regression analysis with TMB as a covariate (Fig. 3a) (Additional file 1: Table S6). After categorization by tumor type, the mutations associated with the non-T cell-inflamed phenotype showed a tumor-specific pattern where individual mutations were enriched in some tumor types but not others (Additional file 2: Fig. S1). Mutation in *IDH1* was found to have the strongest association with the non-T cell-inflamed phenotype across all cancers (FDR-adjusted *P* = 6.75E−16). This mutation was dominantly present in gliomas but also found in other tumor types currently considered non-immunotherapy responsive such as cholangiocarcinoma, breast cancer, and sarcoma. Mutation of *GNAQ* is strongly associated with the non-T cell-inflamed phenotype across tumors and predominately in uveal melanoma, a subset of melanoma known to be resistant to immune-checkpoint blockade [44] (FDR-adjusted *P* = 0.050). Several of the identified genes are highly associated with the non-T cell-inflamed phenotype across cancers and regulate shared or connected signaling pathways. For example, *CTNNB1*, *APC*, and *AXIN1* from the β-catenin pathway cross-talk with PTEN signaling [45] and all have been associated with immune exclusion in our prior work [8]. Mutation in *KDM6A* has not previously been described as mediating the non-T cell-inflamed phenotype however KDM6A is a known epigenetic regulator of interleukin-6 and IFN-β [46] and linked to autoimmunity [47]. Mutations in *FBXW7*, a tumor suppressor that regulates ubiquitin-mediated protein degradation, have been associated with resistance to anti-PD1 in melanoma by reducing MHC class I expression [48]. We used the same significance level cutoff (FDR-adjusted *P* < 0.10) for filtering the genes enriched in the T cell-inflamed phenotype. With TMB adjustment, we observed only three genes associated with the T cell-inflamed phenotype including *VHL*, *PBRM1*, and *CDH10* (Fig. 3b) (Additional file 1: Table S7).

Among identified mutations enriched in non-T cell-inflamed tumors, many are known cancer driver mutations. Figure 3c demonstrates identified somatic mutations in genes including *IDH1* (R132), *CTNNB1* (across exon 3 and K335), *CIC* (R215 and R1512/R1515), and *NRAS* (Q61). To access the functional consequence of the mutations, we used OncoKB annotator to interpret NSSMs from the 29 genes significantly more frequently mutated in non-T cell-inflamed tumors in a pan-cancer fashion (Additional file 1: Table S8). After collapsing identical NSSMs present in different samples, 4693 unique NSSMs (defined by gene name and amino acid alteration) were kept for analysis, including 1089 (23.2%) loss of function (LOF) and 124 (2.6%) gain of function (GOF) somatic mutations. We summarized the distribution of samples harboring NSSMs by mutation effect such as LOF, GOF, switch of function (SOF), neutral (NTR), or unknown (UNK) (Additional file 1: Table S9). The mutations that a gene carries were almost all annotated as GOF or LOF, or SOF (only in *IDH1*). For example, out of 394 samples mutated in *CTNNB1*, 240 (61%) carry GOF mutations, none annotated as LOF, and the rest as unknown. Other genes with predominantly GOF mutations include *FGFR3*, *GNAQ*, *KIT*, *NRAS*, and *PTPN11*. In support of the association of *PTPN11* GOF mutations with the non-T cell-inflamed phenotype, inhibition of PTPN11 (*alias* SHP2) induces anti-tumor immunity and shows higher efficacy when combined with anti-PD1 compared to monotherapy [49]. We note that in genes described in the literature, either LOF or GOF may lead to activation of known downstream target pathways. Among the mutations shown in Fig. 3c, R132 mutations in *IDH1* [50], Q61 mutations in *NRAS* [51], and across exon 3 [52] and K335 mutations [53] in *CTNNB1* were all predicted to be GOF, consistent with the literature. Conversely, R215 and R1512/R1515 mutations in *CIC* are described to be LOF; however, CIC is a known tumor suppressor that upon disruption leads to enhanced MAPK signaling [54]. We repeated the same analysis for the three genes significantly associated with the presence of T cell inflammation. *PBRM1* and *VHL* both carry LOF mutations while *CDH10* mutations were annotated as unknown (Additional file 1: Table S8 and Additional file 1: Table S9).

### Activation of transcriptional programs correlates with the non-T cell-inflamed tumor microenvironment across cancer types

**Fig. 4:**
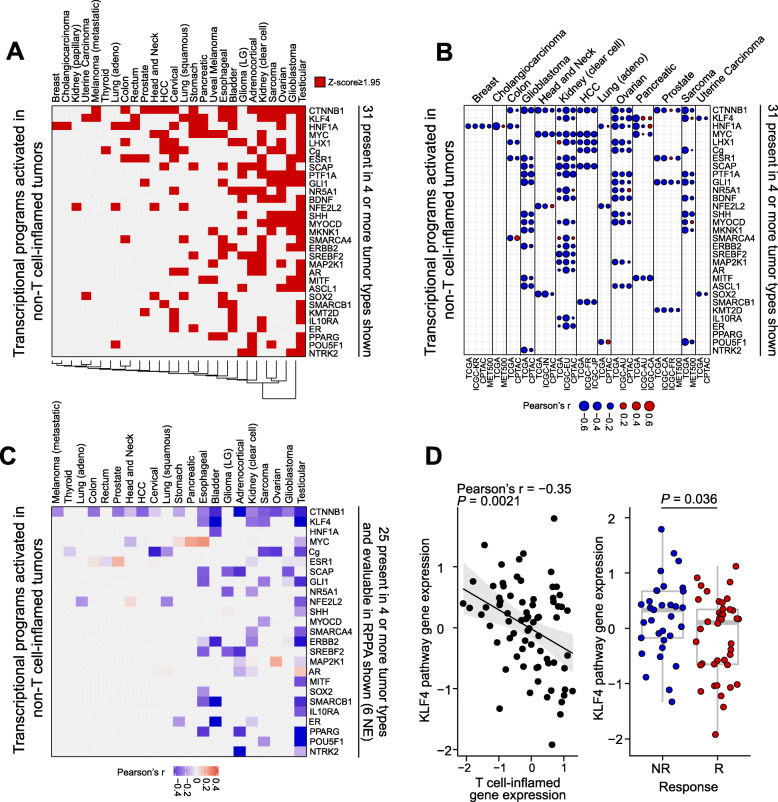
Pan-cancer pathway activation in non-T cell-inflamed relative to T cell-inflamed tumors from RNAseq gene expression. **a** Each tumor type shows transcriptional programs activated in non-T cell-inflamed relative to the T cell-inflamed tumor group. Thirty-one transcriptional programs present in four or more tumor types are shown. Red color labels those at activation *z*-score ≥ 1.95 predicted by IPA causal network analysis (see the “Methods” section). The full list of 266 pathways activated in one or more tumor types is provided in Additional file 1: Table S10. **b** External validation of activated transcriptional programs at gene expression level in three independent cancer genomic databases (ICGC, CPTAC, and MET500). Thirty-one transcriptional programs from **a** are shown on the row, and 13 tumor types with at least one validation cohort are shown on the column. Cohorts from TCGA or independent validation datasets are shown side by side for the same tumor type. The description and sample size of studies from each database are provided in Additional file 1: Table S1. Size of circle represents Pearson’s correlation coefficient *r* of each transcriptional program versus T cell-inflamed gene expression, with blue color indicating negative correlation (Pearson’s *r* < 0) and red color indicating positive correlation (Pearson’s *r* > 0). **c** RPPA validation of activated transcriptional programs at protein level. Twenty-five out of 31 transcriptional programs from **a** are shown. Six do not have corresponding protein in RPPA data, hence not shown (NE = not evaluable). Color represents Pearson’s correlation coefficient *r* of each transcriptional program versus T cell-inflamed gene expression, with blue color indicating negative correlation (Pearson’s *r* < 0), and red color indicating positive correlation (Pearson’s *r* > 0). The full list of correlation statistics is provided in Additional file 1: Table S11. **d** KLF4 pathway activation in a clinical cohort. Only pre-treatment samples are shown (*n* = 73). Left panel: correlation between KLF4 pathway gene expression and T cell-inflamed gene expression. Right panel: KLF4 pathway gene expression in non-responders (NR, including PD and SD) (*n* = 33) and responders (R, including PR and CR) (*n* = 40). IPA’s causal network analysis is used in **a**, Pearson’s correlation is used in **b**, **c**, and the left panel of **d**. Two-sided Welch Two Sample *t*-test was used in the right panel of **d**

Our previous work suggested that activation of transcriptional programs may be another mechanism associated with the non-T cell-inflamed phenotype and only partially overlaps with somatic mutations [8]. Therefore, we performed an unbiased genome-wide pathway discovery following the same protocol as previously described [8]. DEGs were identified between non-T cell-inflamed and T cell-inflamed tumors within each cancer type, and upstream regulators were predicted based on the DEG-encoding target molecules using causal network analysis [26]. Pathways were ranked by the number of tumor types sharing activation of the same pathway (Fig. 4a; all pathways and their activation status in individual cancer type are provided in Additional file 1: Table S10).

Among the top pathways, including 31 activated in at least four tumor types, we observed a strong impact of *CTNNB1* as well as several other pathways described in the literature as influencing anti-tumor immunity such as *MYC* [55], *PPARG* [56] and *SHH* (sonic hedgehog) [57]. In addition, we identified transcriptional programs correlated with the non-T cell-inflamed phenotype such as *KLF4*, *HIF1A*, *KMT2D* (*alias MLL2*), *NFE2L2* (*alias NRF2*), androgen receptor (AR), and estrogen receptor (ER) signaling, which have some literature support for modulating the immune response [42, 58, 59].

To further investigate the inverse correlation between the T cell-inflamed gene expression and the activated pathways, we repeated the same analysis in independent tumor-type specific disease cohorts from three external databases (ICGC [15], CPTAC [16, 17], and MET500 [18, 19]) covering 13 tumor types. For each of the top 31 pathways previously discovered (Fig. 4a), we observed high consistency between TCGA and other databases in the relevant tumor type (Fig. 4b). We calculated the similarity between the cohorts observing Jaccard similarity coefficient of 0.92 in ICGC, 0.75 in CPTAC, and 0.81 in MET500 (see the “Methods” section). When analyzing all 266 activated pathways in the 13 tumor types, we continued to observe a high concordance with Jaccard similarity coefficient (ICGC, 0.88; CPTAC, 0.76; MET500, 0.77), as shown in Additional file 2: Fig. S2.

To validate the activation of transcriptional programs at the protein level, we correlated the normalized RPPA protein abundance of the predicted upstream regulator and/or aggregation of the downstream target molecules with the T cell-inflamed gene expression for each tumor type within TCGA. Out of 266 activated pathways from 26 tumor types (531 total), 262 (49.3%) have corresponding proteins measured on the RPPA platform and were included in the correlation analysis (25 shown in Fig. 4c). Out of 262 correlations, 242 (92.4%) showed anti-correlation between the pathway protein level and the T cell-inflamed gene expression (Pearson’s correlation coefficient *r* < 0). One hundred and seventy (64.9%) reached a significance level of 0.05 after FDR-correction for multiple testing. A complete listing of RPPA correlations is provided in Additional file 1: Table S11.

To explore the potential clinical relevance of pathway activation on checkpoint immunotherapy, we analyzed pathway gene expression in correlation with treatment response from a previously published metastatic melanoma cohort with RNAseq data available [20, 21] (see the “Methods” section). We included the 73 pre-treatment samples in analysis, given the untreated nature of the metastatic melanoma cohort of TCGA. Focusing on KLF4, a component of the sonic hedgehog signaling pathway noted to be activated in metastatic melanoma from TCGA, we confirmed the anti-correlation between T cell-inflamed gene expression and pathway gene expression (*P* = 0.002, Pearson’s correlation; Fig. 4d left panel). KLF4 pathway gene expression was also found to be significantly higher in non-responders relative to responders (*P* = 0.036, two-sided Welch Two Sample *t*-test; Fig. 4d right panel). Though we found an inconsistent association of WNT/β-catenin with treatment response, a growing body of evidence supports WNT/β-catenin activation with immunotherapy resistance [60–63].

### Mutations or pathways associated with the non-T cell-inflamed phenotype co-occur within individual tumors

**Fig. 5:**
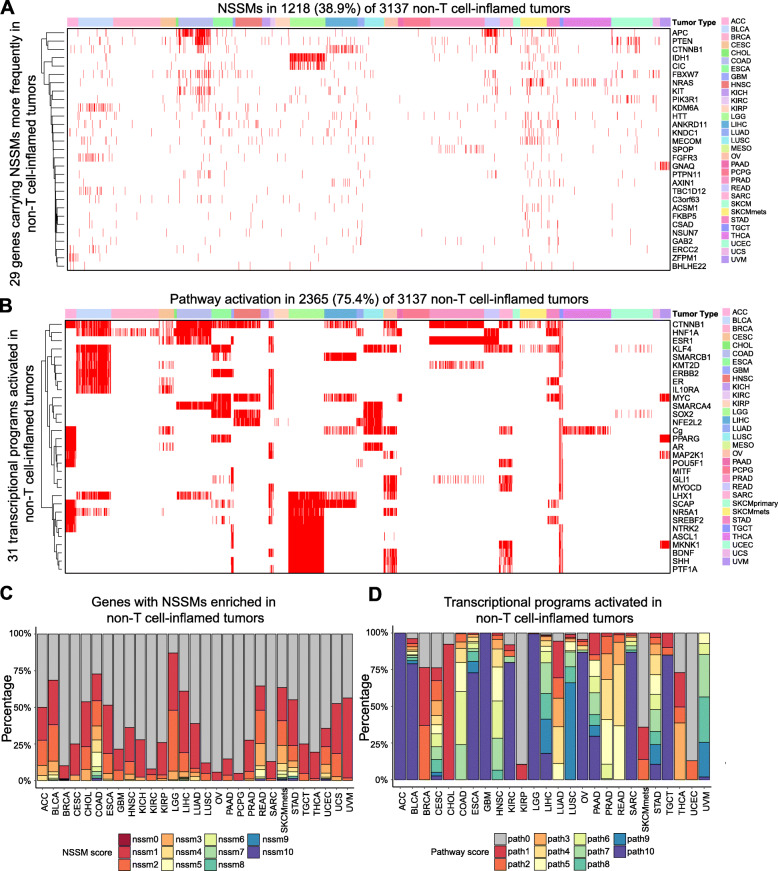
Multiple mutation- or pathway-mediated resistance mechanisms co-occur in non-T cell-inflamed tumors. **a** Co-occurrence of NSSMs in 29 genes from Fig. 3a at per patient level. Annotation bar above the heatmap indicates tumor type. **b** Co-activation of 31 transcriptional programs from Fig. 4a at per patient level. Annotation bar above the heatmap indicates tumor type. *n* = 3137 non-T cell-inflamed tumors are shown in **a** and **b**. The full list of co-mutated genes or co-activated pathways at per patient level is provided in Additional file 1: Table S12. **c** Percentage of tumor samples with one or more genes carrying NSSMs in the same patient from non-T cell-inflamed tumor group. NSSM score categories 0–10 are shown. Samples with more than 10 co-activated genes were collapsed into category 10. **d** Percentage of tumor samples with one or more transcriptional programs activated in the same patient from non-T cell-inflamed tumor group. Pathway score categories 0–10 are shown. Samples with more than 10 co-activated pathways were collapsed into category 10. The sample percentages and distance metrics are provided in Additional file 1: Table S14

To investigate the interaction between somatic mutations and/or activated transcriptional programs, we summarized the number of mutations or activated pathways on the per-patient level. Out of 3137 patients from the non-T cell-inflamed tumor group, 1218 (38.8%) harbor NSSMs (Fig. 5a) in at least one of the 29 non-T cell-inflamed genes from our pan-cancer analysis above and 2365 (75.4%) show activation in at least one of the 266 transcriptional programs from those reported above (31 activated in 4 or more tumor types shown in Fig. 5b). Taking together, 2572 (82.0%) across cancer types demonstrate evidence of at least one mutation or pathway activation (per-patient mutated genes or activated pathways shown in Additional file 1: Table S12; summary statistics shown in Additional file 1: Table S13).

To better understand the heterogeneity of mutations or pathway activation in non-T cell-inflamed tumors across different tumor types, we assigned an NSSM score (defined as, the number of co-mutated genes per sample) and a pathway score (defined as, the number of co-activated pathways per sample) for each tumor. We calculated the percentage of samples for each tumor type harboring increasing NSSM score or pathways score on a continuous scale (Fig. 5c, d). We compared the distribution of samples with co-mutated genes or co-activated pathways across tumor types, observing significant differences (*P* = 8.60E−08, *D* = 0.81, two-sided two-sample Kolmogorov-Smirnov test), indicating greater heterogeneity among different tumor types for pathway activation than for mutations (defined by, the deviation of the distance distribution in each tumor type from the average distribution across all tumor types; see the “Methods” section and Additional file 2: Fig. S3 for the methodology to quantify and compare such heterogeneity as well as Additional file 1: Table S14 for the data). We observed extreme outliers in pathway activation such as cholangiocarcinoma (CHOL) with only one pathway (HIF1A) activated in 12 out of the 13 non-T cell-inflamed tumors (92.3%), as compared to glioblastoma (GBM) with 10 or more pathways co-activated in 14 out of the 14 non-T cell-inflamed tumors (100%) (*P* = 1.035E−07, two-sided Fisher’s exact test).

**Fig. 6:**
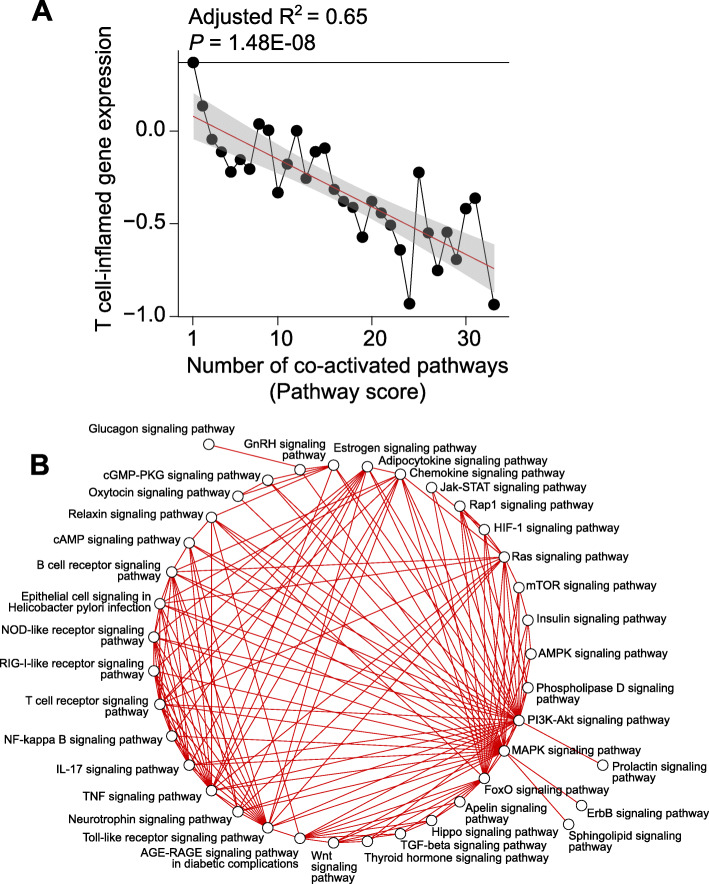
Interactions between co-activated pathways and T cell-inflamed gene expression. **a** Relationship between T cell-inflamed gene expression and increasing pathway scores. Each dot represents the median expression of the T cell-inflamed gene signature (shown on the *y*-axis) across samples in the score category (shown on the *x*-axis). Score categories with ≥ 10 samples were included in the analysis. Red line shows the linear regression model fitted with the observed data points (see the “Methods” section). Gray area indicates the 95% confidence interval. Adjusted *R*^2^ and *P* value are shown above each panel. The complete data and the regression models are provided in Additional file 1: Table S15. **b** Pathway network analysis shows connections between multiple transcriptional programs activated in non-T cell-inflamed tumors from one or more tumor types

Observing the association of individual mutated genes or activated pathways with non-T cell-inflamed tumors, we hypothesized that tumors with increasing numbers of molecular aberrations would be more likely to be non-T cell-inflamed. We analyzed via linear regression the expression of T cell-inflamed gene signature based on the number of co-occurring mutated genes, as above, observing no significant association (*P* = 0.82; data and model provided in Additional file 1: Table S15). We performed a similar analysis using the number of co-occurring activated pathways and detected a significant inverse association between T cell-inflamed gene signature and pathway score (*P* = 1.48E−08; Fig. 6a; data and model provided in Additional file 1: Table S15). We also tested polynomial regression models, but the fitting was not significantly better than linear regression (*P* > 0.05, likelihood ratio test).

Cognizant of cross-talk between canonical signaling pathways, we sought to investigate the connections between activated transcriptional programs as related to the non-T cell-inflamed tumor phenotype. To pursue this, upstream regulator encoding genes were annotated with KEGG pathways and built into a network with nodes representing each KEGG pathway and edges representing the shared molecules between pathways. KEGG pathways were observed to be highly connected including PI3K-Akt signaling, MAPK signaling, and FoxO (Forkhead Box O) signaling (Fig. 6b).

To facilitate the clinical translation of these results, we were interested in identifying existing drugs that might be repurposed as immunomodulatory agents. To pursue this, we queried The Drug Gene Interaction Database [36] (DGIdb), an ensemble database that integrates 20 different resources consisting of existing United States Food and Drug Administration (FDA) drugs. The majority of the relevant molecular mechanisms identified in our study was found to have one or more potentially relevant drugs available (Additional file 1: Table S16). Inhibitors were identified for mutated genes and signaling pathways activated in non-T cell-inflamed tumors, such as FGFR3, IDH1, NRAS, PTEN, AR, MAP 2K1, and NTRK2 which may nominate therapeutic approaches to overcome the non-T cell-inflamed phenotype. Alternatively, well-known agents targeting IFNG, IL2, or TLR9 might be considered as adjuncts in tumor types already demonstrating the T cell-inflamed phenotype.

To provide more context of the findings in this study, we have performed a literature review on the genes associated with non-T cell-inflamed tumors or T cell-inflamed tumors from our pan-cancer analysis. We collected three levels of evidence from the literature, including the mechanism and/or pathway associations of a gene (level 1), role in immune response from non-cancer studies (level 2), and role in tumor immune microenvironment and/or cancer pathogenesis (level 3). We prioritized experimental evidence in our review and only included in silico analysis results if data are from patients treated with checkpoint immunotherapy. The result is provided in Additional file 1: Table S17.

## Discussion

The T cell-inflamed tumor microenvironment, characterized by TIL and evidence for IFN-γ linked adaptive immunity, has been strongly associated with clinical response to checkpoint immunotherapy [39, 64]. Based on our prior observations surrounding the impact of dysregulated oncogenic signaling from the WNT/β-catenin pathway, we performed a per tumor type and pan-cancer analysis to assess the landscape of somatic and transcriptomic events associated with the T cell-inflamed or non-T cell-inflamed phenotype. We stratified solid tumors of TCGA by T cell-inflamed, intermediate, and non-T cell-inflamed status and interrogated genomic aberrations as well as gene expression patterns associated with these phenotypes controlling for TMB. We identified groups of somatic mutations strongly linked to T cell-inflamed or non-T cell-inflamed tumors including some in well-known genes and as well as others that are less well understood. Similarly, we have identified pathways associated with these phenotypes with some established as immuno-suppressive and others previously unknown.

A growing list of tumor mutations has been described which mediate immune exclusion by multiple mechanisms. Taken together, these somatic events broadly lead to a state of decreased innate immunity, antigen presentation, and T cell trafficking to tumors. For example, mutation of *STK11* has been strongly associated with a lack of response to checkpoint inhibitors [43], and mechanistic studies have linked this aberration to suppression of STING agonism [65]. Similarly, MYC activation is known to be reversibly associated with a lack of response to immunotherapy [66] and hypoxic environments [67], commonly associated with liver metastases which are less responsive to immunotherapy [68]. These observations suggest that the progressive spectrum of mutations in cancer may as much contribute to immune exclusion as a direct growth advantage for malignant clones.

In our TMB-adjusted tumor type-specific and pan-cancer analysis, we observed the presence of several mutations that deserve further interrogation on a mechanistic level as driving the non-T cell-inflamed phenotype. For example, the histone demethylase encoded by KDM6A, where gene deletion has long been associated with the Kabuki immunodeficiency syndrome [69], is also known to be associated with immune relevant processes including the type I interferon [46] and hypoxia response [70]. In metastatic melanoma, we observed ITGAX (*alias CD11c*), a marker expressed on myeloid cells with a well-known impact on classical and monocytic dendritic cell function [71], is more frequently mutated in non-T cell-inflamed tumors. Similarly, CIC (Capicua), dominantly mutated in low-grade glioma, is a transcriptional repressor of MAPK [72], and MAPK activation has been linked to an immunosuppressive environment in multiple histologies [73]. We would specifically recommend nuance and further validation for genes that are very commonly mutated in cancer, such as *TP53*, as they appear as extreme outliers in our analysis and yet have some literature support for an association with tumor type-specific immune phenotypes.

As opposed to tumor-intrinsic oncogenic mutations driving immuno-suppression, we have additionally identified recurrent somatic events that may potentiate the anti-tumor immune response. The most obvious of these, *PBRM1*, has previously been shown from patient samples in renal cell carcinoma to impact outcomes to checkpoint blockade [74, 75] and has been demonstrated to augment T cell-mediated killing [76]. We would note that including TMB as a covariate had a substantial impact on the list of genes associated with T cell-inflamed tumors (> 200 identified without adjustment versus three with adjustment) such that only *VHL*, *PBRM1*, and *CDH10* remained significant after FDR correction (FDR-adjusted *P* < 0.10). We were interested to observe that *LRP1B* was the fifth most commonly mutated gene in T cell-inflamed tumors (FDR-adjusted *P* = 3.38E−06) without TMB adjustment; however, this gene was not significant upon TMB adjustment. *LRP1B* mutations have been associated with treatment response to immune-checkpoint blockade independent of tumor type [77]. This suggests to us that further relevant genomic biology may exist within the cadre of mutations associated with T cell-inflamed tumors, though further approaches will be necessary to differentiate them from random mutations in the context of increasing TMB. Moreover, novel tumor-intrinsic immune-related functions for well-known and less well-known genes are increasingly being identified suggesting that anti-tumor immunity is a complicated interaction of host and somatic genomics. Our findings provide a compendium of genes and pathways that deserve further study as potential actionable molecular nodes that could modify or enhance anti-tumor immune responses.

In addition to somatic mutations associated with immune phenotypes, our transcriptional analysis of gene programs associated with non-T cell-inflamed tumors has nominated multiple immediate and novel therapeutic approaches. Our analysis of three independent databases (ICGC, CPTAC, and MET500) confirms these findings as broadly applicable in multiple tumor types highlighting potential clinical relevance and suggesting that integrating some of these molecular pathways deserve investigation as combination approaches with immunotherapy. Examples could include hedgehog signaling in ovarian cancer and hormone receptor (estrogen and androgen) in multiple diseases including urothelial or adrenocortical cancers. We also would hypothesize that the T cell-inflamed versus non-T cell-inflamed phenotype in certain tumor types may be particularly amendable to this approach given fewer numbers of pathways co-activated in the same tumor sample. For example, HIF1A is the only pathway activated in over 90% of the non-T cell-inflamed tumors in cholangiocarcinoma, perhaps emphasizing it as a therapeutic target.

While we and others have previously described individually dominant transcriptional patterns linked to immunosuppression, the pan-cancer analysis we provide here argues that transcriptional programs that are substantially shared across tumor types. We observe that tumors with increasing numbers of co-activated pathways are progressively less T cell-inflamed, suggesting at least an additive impact on tumor immune exclusion. We also see CTNNB1, KLF4, HIF1A, and MYC as particularly important across many cancers as well as evidence to suggest substantial cross-talk and perhaps overlap. Because of this, we were interested to investigate the interactions of these pathways and noted MYC signaling as perhaps the most important node associating with a lack of the T cell-inflamed phenotype. Consideration of approaches to alleviate MYC related immuno-suppression should be a high priority.

While targeting of some oncogenic pathways remains difficult, our analysis has identified multiple targets for which translation is possible with immediate examples surrounding the PI3K, MAPK, and FGFR pathways. We note that the identified mutations in these pathways commonly appear to be the same as those for which small molecule targeted inhibitors have already been developed. Especially targeting of sonic hedgehog signaling in multiple tumors might be prioritized. Tumor secreted sonic hedgehog has been shown to be immunosuppressive. This can be overcome by smoothened inhibition, with enhanced anti-cancer effect in combination with anti-PD1 [78]. Beyond these, we have particularly focused on IDH1 as a metabolism mediator of immunosuppression. Based on our data in concert with functional validation from human and murine models [79], we have taken forward a phase II clinical trial of the IDH1 inhibitor ivosidenib with anti-PD1 in patients selected by *IDH1* mutation (NCT04056910). More broadly, our FDA database analysis suggests that there are many drugs with the potential to be re-evaluated, repurposed as immune modifiers potentially to combine with immunotherapy.

Noting that some of the findings from our analysis already have confirmation from clinical trials, we do acknowledge limitations of this work. Using the T cell-inflamed gene expression phenotype is an imperfect approximation of patient response in clinical trials however interferon associated gene expression does appear to perhaps be the best translational biomarker currently available. Despite failure of TMB as a selection marker in multiple phase III clinical trials, it has become integrated into clinical practice and we have incorporated it as a covariate in our analyses here. Additionally, our analysis of TCGA, ICGC, CPTAC, and MET500 does not facilitate specific outcomes to immunotherapy treatment response though preliminary analysis of patients treated with anti-PD1 suggests that these findings may impact outcomes. Using large dataset additionally does allow a large discovery power to identify novel genes and pathways associated with immune exclusion.

## Conclusions

Treatment with cancer immunotherapy has become a backbone of cancer therapy however is ineffective in the majority of patients and novel biomarkers as well as molecular targets are needed to advance the field. In an unbiased analysis, we have identified molecular alterations already validated from patient tissues but more importantly many somatic and transcriptional aberrations that deserve immediate study. Based on these data, we have already advanced to clinical trials and believe that many drugs are available in the public domain that might be considered for immunotherapy repurposing. As other biomarkers or novel insights (i.e., epigenetics) become more robust, it may be possible to integrate this data into similar analyses to identify further mediators of immunotherapy response and resistance.

## Supplementary information

Additional file 1: **Table S1.** List of datasets used in this study (TCGA, external cancer genomic databases (ICGC, CPTAC, and MET500), and a cohort of patients treated with anti-PD1 from a published study). **Table S2.** List of cancer ID and description from TCGA included in this study. **Table S3.** List of the 160 genes from the T cell-inflamed expression signature. **Table S4.** List of mutated genes investigated in the individual cancer type analysis that are relatively enriched in non-T cell-inflamed relative to T cell-inflamed tumors. All genes are shown without filtering by *p*-values. **Table S5.** List of mutated genes investigated in the individual cancer type analysis that are relatively enriched in T cell-inflamed relative to non-T cell-inflamed tumors. All genes are shown without filtering by p-values. **Table S6.** List of mutated genes investigated in the pan-cancer analysis that are relatively enriched in non-T cell-inflamed relative to T cell-inflamed tumors. All genes are shown without filtering by p-values. **Table S7.** List of mutated genes investigated in the pan-cancer analysis that are relatively enriched in T cell-inflamed relative to non-T cell-inflamed tumors. All genes are shown without filtering by p-values. **Table S8.** Annotation of mutation effects (LOF, GOF, etc.) for NSSMs from genes associated with non-T cell-inflamed phenotype (*n* = 29, from Fig. 3a) and genes associated with the T cell-inflamed phenotype (*n* = 3, from Fig. 3b). **Table S9.** Distribution of tumor samples carrying NSSMs in genes associated with non-T cell-inflamed phenotype (n = 29, from Fig. 3a) or genes associated with the T cell-inflamed phenotype (n = 3, from Fig. 3b) by mutation effect categories (LOF, GOF, etc.). **Table S10.** List of 266 pathways predicted to be activated in non-T cell-inflamed relative to T cell-inflamed tumors. 1 = activated; 0 = not-activated. **Table S11.** RPPA validation of activated transcriptional programs at protein level. **Table S12.** List of genes carrying NSSMs enriched in non-T cell-inflamed tumors (n = 29, from Fig. 3a) or transcriptional programs activated in non-T cell-inflamed tumors across at least 4 cancer types (*n* = 31, from Fig. 4a) at per patient level. **Table S13.** Summary of samples carrying NSSMs enriched in non-T cell-inflamed tumors (n = 29, from Fig. 3a) or transcriptional programs activated in non-T cell-inflamed tumors (*n* = 266). **Table S14.** Heterogeneity analysis of the NSSM and pathway score distribution across different tumor types. **Table S15.** Linear regression model of the T cell-inflamed gene expression and NSSM or pathway scores. **Table S16.** List of drugs targeting relevant molecular mechanisms identified in our study from The Drug Gene Interaction Database (DGIdb). **Table S17.** Three-level literature review of the genes associated with the non-T cell-inflamed phenotype (n = 29, from Fig. 3a) and genes associated with the T cell-inflamed phenotype (n = 3, from Fig. 3b). (XLSX 22 mb)

Additional file 2: **Fig. S1.** Pan-cancer distribution of mutations from 29 genes associated with non-T cell-inflamed phenotype, colored by cancer type. **Fig. S2.** External validation of activated transcriptional programs at gene expression level in three independent cancer genomic databases (ICGC, CPTAC, and MET500). **Fig. S3.** Methodology to quantify and compare heterogeneity of distribution of samples carrying NSSM scores or pathway scores across different tumor types. (PDF 490 kb)

## Data Availability

The original data files were downloaded from TCGA, ICGC, and CPTAC and publicly available on Broad’s GDAC-Firehose website [14], ICGC data portal [15, 16], and GDC data portal [17]. The MET500 RNAseq data in FastQ format are under controlled access in dbGAP (accession number phs000673.v4.p1) [18]. The metastatic melanoma patient cohort treated with anti-PD1 RNAseq data in FastQ format is publicly available in SRA (accession number PRJEB23709) [20, 21]. Preprocessed data files are provided as Additional file 1. Per-gene per-tumor type mutation frequency table and *TP53* mutation annotation data files were deposited on a publicly accessible GitHub Repository [28] (https://github.com/riyuebao/T-cell-inflamed-multicorrelates).

## Abbreviations

TMB: Tumor mutational burden
CPTAC: Clinical Proteomic Tumor Analysis Consortium
MET500: 500 cancer patients diagnosed with metastatic cancers burden
BH: Benjamini-Hochberg
FDR: False discovery rate
GDC: Genomic Data Commons
PD: progressive disease
SD: stable disease
PR: partial responder
CR: complete responder
CYT: immune cytolytic activity
GEP: interferon gene expression profile
TIL: Tumor-infiltrating lymphocytes
IFN: Interferon
PD1: programmed cell death protein 1
TCGA: The Cancer Genome Atlas
TCGA-CDR: TCGA Pan-Cancer Clinical Data Resource
RSEM: RNA-Seq by Expectation Maximization
CPM: Counts per million
DEGs: Differentially expressed genes
limma: Linear Models for Microarray and RNA-Seq Data
KEGG: Kyoto Encyclopedia of Genes and Genomes
SNVs: Single nucleotide variants
Indels: Insertions and deletions
NSSMs: Non-synonymous somatic mutations
TMM: Trimmed mean of *M* values
ICGC: International Cancer Genome Consortium
RPPA: Reverse phase protein array
DGIdb: Drug Gene Interaction Database
GOF: Gain of function
LOF: Loss of function
FDA: Food and Drug Administration
