# Supplementary material for: Molecular correlates and therapeutic targets in T cell-inflamed versus non-T cell-inflamed tumors across cancer types
Source: Genome Med. 2020 Oct 27;12:90. doi: 10.1186/s13073-020-00787-6 (PMC7590690; doi:10.1186/s13073-020-00787-6)
Supplement: Supplementary file 2 — Fig. S1. Pan-cancer distribution of mutations from 29 genes associated with non-T cell-inflamed phenotype, colored by cancer type. Fig. S2. External validation of activated transcriptional programs at gene expression level in three independent cancer genomic databases (ICGC, CPTAC, and MET500). Fig. S3. Methodology to quantify and compare heterogeneity of distribution of samples carrying NSSM scores or pathway scores across different tumor types. (PDF 490 kb) [file 13073_2020_787_MOESM2_ESM.pdf]

Supplementary Materials

for

Molecular correlates and therapeutic targets in T cell-inflamed versus non-T cell-inflamed tumors across cancer types

Running Title: Molecular correlates of inflamed or non-inflamed tumors

Authors: Riyue Bao[1,2], Daniel Stapor[2], Jason J. Luke[1,2,*]

[1]Hillman Cancer Center, UPMC, Pittsburgh, PA

[2]Department of Medicine, University of Pittsburgh, Pittsburgh, PA

[*]**Corresponding Author:**

Jason J. Luke, MD, FACP

Associate Professor of Medicine

University of Pittsburgh Medical Center and Hillman Cancer Center

5150 Centre Ave. Room 564

Pittsburgh PA 15232

Telephone: (412) 647-2811

Fax: (412) 623-7948

lukejj@upmc.edu

Fig. S1 to S3 are included in this file.

Tables S1 to S17 are provided as a separate spreadsheet.

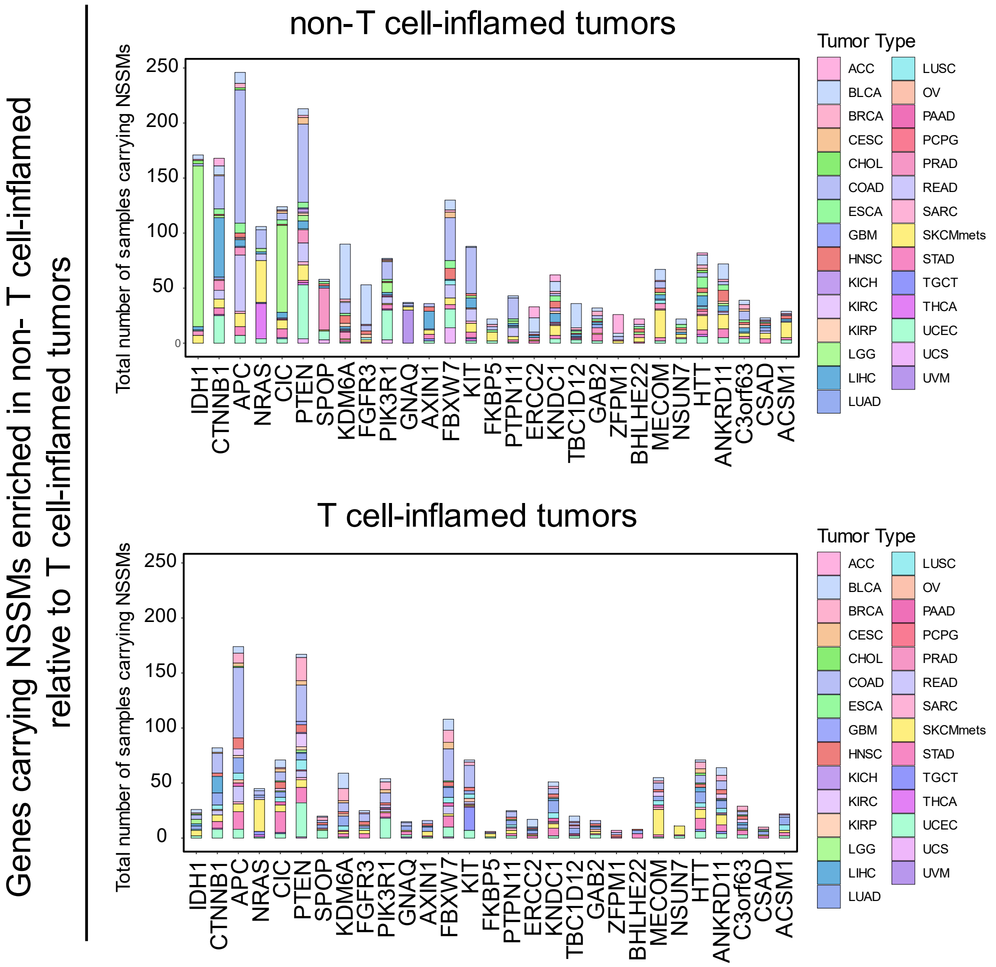

Fig. S1. Pan-cancer distribution of mutations from 29 genes associated with non-T cell-inflamed phenotype, colored by cancer type. Genes are listed on the x-axis, and the number of tumors carrying NSSMs is shown on the y-axis. Each color represents individual cancer types. Cancer ID and description can be found in **Additional file 1: Table S1**.

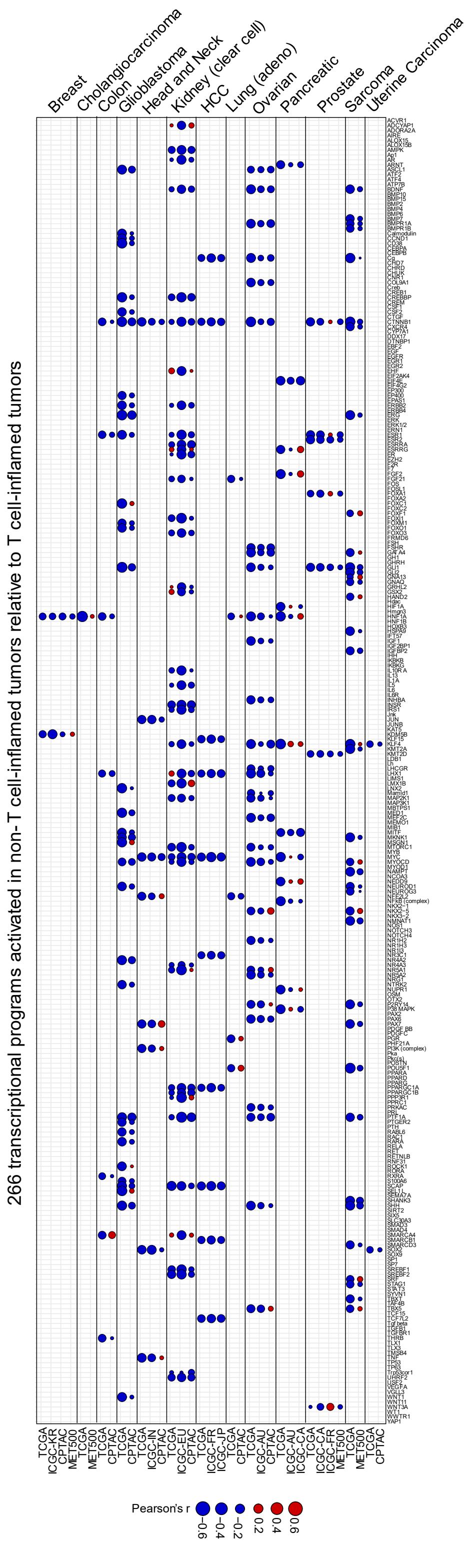

Fig. S2. External validation of activated transcriptional programs at gene expression level in three independent cancer genomic databases (ICGC, CPTAC, and MET500). Two

hundred sixty-six transcriptional programs activated in non-T cell-inflamed tumors relative to T cell-inflamed tumors are shown on the row, and 13 tumor types with at least one validation cohort are shown on the column. Cohorts from TCGA or independent validation datasets are shown side by side for the same tumor type. The description and sample size of studies from each database are provided in **Additional file 1: Table S3**. Size of a circle represents Pearson's correlation coefficient r of each transcriptional program versus T cell-inflamed gene expression, with blue color indicating negative correlation (Pearson's $r < 0$), and red color indicating positive correlation (Pearson's $r > 0$).

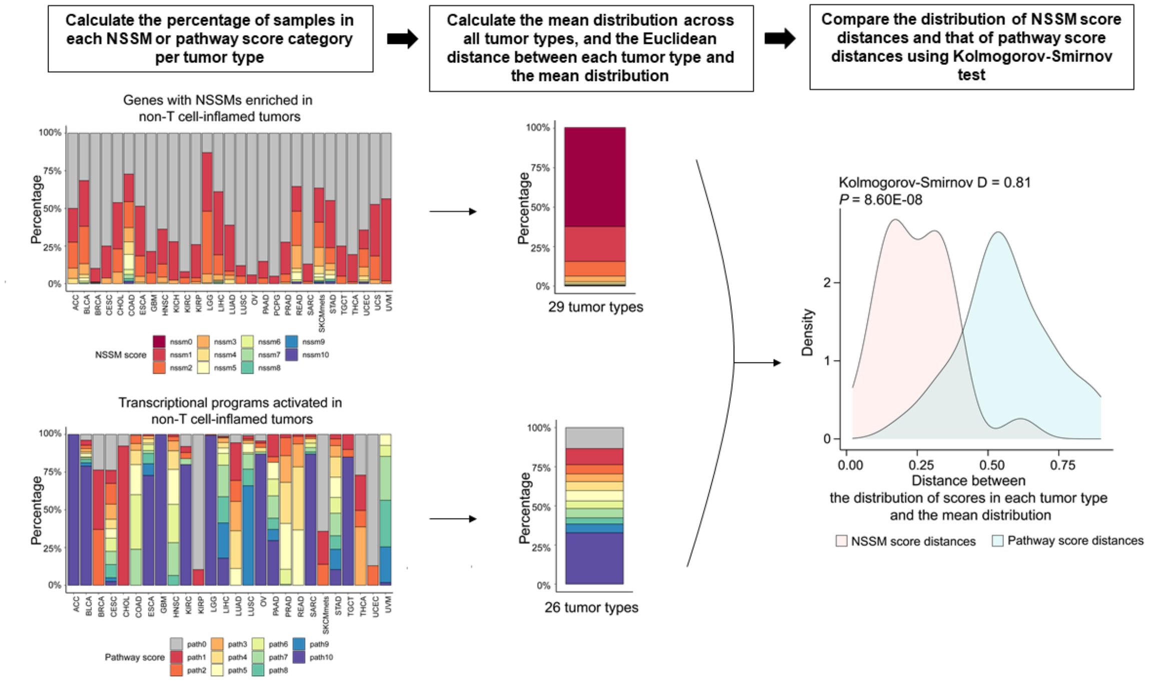

Fig. S3. Methodology to quantify and compare heterogeneity of distribution of samples carrying NSSM scores or pathway scores across different tumor types. Three steps were involved. (Step 1) The percentage of samples in each NSSM or pathway score category in the non-T cell-inflamed tumors was calculated per tumor type (**Fig. 5C** and **5D** were re-included here for illustration purposes of the method). (Step 2) The mean distribution of NSSM score categories was calculated across all tumor types. For each tumor type, the Euclidean distance was computed between the distribution of NSSM or pathway scores in this tumor type and the mean distribution (hereafter referred to as, the NSSM or pathway score distances). (Step 3) The NSSM score distances and pathway score distances were compared to test if those are from the same distribution using two-sided two-sample Kolmogorov-Smirnov test. Significant differences indicated that the distribution of NSSM score distances was different from that of the pathway score distances, further suggesting different degrees of heterogeneity among tumor types between mutations and pathways. See **Methods** for a detailed description of the analysis procedure with relevant data provided in **Additional file 1: Table S14.**
